# Methyl-CpG binding protein 2 is associated with the prognosis and mortality of elderly patients with hip fractures

**DOI:** 10.1016/j.clinsp.2022.100034

**Published:** 2022-04-15

**Authors:** Xuejian Gao, Shan Xue, Fuqiang Yang, Baoling Wu, Xiaojing Yu, Baoquan An

**Affiliations:** Department of Orthopedics, The 80^th^ Army Hospital of Chinese People's Liberation Army, Weifang, Shandong, China

**Keywords:** MECP2, Prognosis, Hip fracture, MECP2, Methyl-CpG binding Protein2, IL, Interleukin, ELISA, enzyme linked immunosorbent assay, BI, Bar-thel Index, TNF-α, tumor necrosis factor-α

## Abstract

**Objectives:**

To investigate the expression level and clinical significance of Methyl-CpG binding Protein 2 (MECP2) in elderly patients with hip fractures.

**Methods:**

This prospective observational study included 367 elderly patients with hip fractures between April 2016 and December 2018. All the patients were treated with internal fixation or joint replacement. In addition, 50 healthy elderly individuals were enrolled as healthy controls. The serum levels of MECP2 and inflammatory factors Interleukin (IL)-1β, IL-6, IL-8, and Tumor Necrosis Factor (TNF)-α was determined by enzyme-linked immunosorbent assay. Data on patients' basic characteristics and postoperative complications were collected. The Harris score was used to assess hip function at 1-month, 3-months, and 6-months after surgery. Patient quality of life was measured using the Barthel Index (BI) score 3-months after surgery. The 1-year mortality was analyzed using the Kaplan-Meier curve, and logical regression was used to analyze the risk factors for mortality.

**Results:**

No significant differences were observed in the basic clinical characteristics of all patients. The serum MECP2 levels were remarkably high in patients with hip fractures and negatively correlated with serum IL-1β, IL-6, and TNF-α levels. Patients with higher MECP2 predicted higher dynamic Harris scores, lower postoperative complications, lower 1-year mortality, and higher BI scores. Logical regression showed that age was the only independent risk factor for postoperative 1-year mortality in elderly patients with hip fractures.

**Conclusion:**

Lower MECP2 predicted poor prognosis and higher 1-year mortality in elderly patients with hip fractures.

## Highlights

• MECP2 was downregulated in elderly patients with hip fracture.
• MECP2 was correlated with inflammatory factors in hip fractures.
• Lower MECP2 predicted poor clinical outcomes of hip fractures.
• Lower MECP2 predicted higher mortality and postoperative complications.

## Introduction

With the trend of population aging, related diseases, including fractures, are increasingly common, with an increasing incidence in many regions.^1^^,^^2^ Hip fractures account for the majority of osteoporotic fragility fractures, and more than 40% of the burden of osteoporosis worldwide is caused by hip fractures.^3^ For elderly patients, a hip fracture may lead to morbidity, mortality, and high costs of health and social care,^4^^,^^5^ and a deeper understanding of the risk factors for the prognosis of hip fracture is still needed.

Methyl-CpG binding Protein 2 (MECP2) is a protein that can bind methylated deoxyribonucleic acid and is involved in many diseases such as seizures,^6^ Rett's syndrome,^7^ and chronic pain.^8^ It has been observed that MECP2 can suppress inflammation, which is considered a risk factor for hip fractures.^9^^,^^10^ In recent decades, MECP2 was also found to be associated with bone formation.^11^ However, few studies have focused on the clinical significance of MECP2 in hip fractures.

In the present study, the authors demonstrated that lower expression of MECP2 was correlated with a poor prognosis in patients with hip fractures, which might provide evidence for the role of MECP2 in the process of hip fractures.

## Materials and methods

### Patients

The present prospective observational study included 367 elderly patients with hip fractures who were admitted to the authors’ hospital between April 2016 and December 2018. All patients were consecutively enrolled in this study. The inclusion criteria were as follows: 1) Patients ≥ 65 years of age; 2) Patients were radiographically diagnosed with femoral neck or intertrochanteric fractures; 3) Patients were American Society of Anesthesiologists (ASA) stage I‒II; 4) Patients were hospitalized within 48h after the fracture. The exclusion criteria were as follows: 1) Patients with other fractures; 2) Pathological hip fractures caused by a tumor; 3) Patients with mental diseases such as schizophrenia; 4) Patients with severe renal, liver, or cardiovascular diseases, such as severe diabetic nephropathy, heart failure, hepatitis, and other surgical contraindications. Additionally, 50 healthy elderly individuals who underwent routine physical examinations were enrolled as healthy controls. All healthy individuals underwent routine physical examination and were confirmed as not having any diseases that might have influenced the study. This study was approved by the ethics committee of the 80^th^ Army Hospital of the Chinese People's Liberation Army.

For surgical methods, non-displaced femoral neck fractures and simple intertrochanteric fractures were mainly treated by internal fixation, including hollow screw fixation and dynamic hip screw fixation. For displaced femoral neck fractures and severely displaced and comminuted intertrochanteric fractures, total hip replacement or artificial femoral head replacement was selected according to the patient's condition. All implants were cemented. All surgical procedures were routine and were conducted by the same surgical team according to the same protocol.

### Serum levels of MECP2 and inflammatory factors

Blood samples from all patients were collected within 24h after admission before undergoing surgery. Serum levels of MECP2 and inflammatory factors Interleukin (IL)-1β, IL-6, IL-8, and Tumor Necrosis Factor (TNF)-α were determined by Enzyme-Linked Immunosorbent Assay (ELISA) using commercially available ELISA kits according to the manufacturer's instructions.

### Data collection and measurement

Demographic data, including age, sex, Body Mass Index (BMI), and clinical data, including the surgical type, ASA stage, fracture type, preoperative complications, and postoperative complications, were collected. For hip function, the Harris score was used before surgery and at 1-month, 3-months, and 6-months after surgery. The Barthel Index (BI) score was used to measure patient quality of life 3-months after surgery. For survival analysis, the survival duration was defined as the time from hospital admission to death or the last follow-up. All patients were followed up for 1-year. Patients who were lost to follow-up were excluded.

### Statistical analysis

The measurement data are expressed as the mean ± standard deviation. The Chi-Square analysis was used to compare rates. Comparisons between two groups were conducted using Student's *t*-test. The Kaplan-Meier (K-M) curve was used for 1-year survival analysis. Pearson's correlation assay was used for the correlation analysis. Binary regression analysis was used to analyze 1-year mortality risk factors using a back step. Differences were considered statistically significant when the p-value was < 0.05. All calculations were performed using SPSS version 20.0.

## Results

### Basic characteristics of all patients

Among all patients, the mean age was 79.64±9.34 years, with 163 males and 204 females. In terms of fracture type, 231 (62.94%) patients had femoral neck fractures, and 136 (37.06%) had intertrochanteric fractures. The surgical strategies were simple fractures with fixation for 80 (21.80%) patients, complex fractures with fixation for 76 (20.71%) patients, simple fractures with arthroplasty for 112 (30.52%) patients, and complex fractures with arthroplasty for 99 (26.98%) patients. No significant difference was observed in the basic characteristics of age, sex, and BMI between the patients and healthy controls (Table 1).

**Table 1:** Basic characteristics of all patients and participants.

Variables	Hip fracture, (n = 367)	Healthy controls, (n = 50)	p
Age, year	79.64±9.34	78.90±9.71	0.601
Sex, female (%)	204 (55.59)	27 (54.00)	0.821
BMI, kg/m^2^	21.57±1.99	21.39±1.90	0.544
Fracture type, n (%)		‒	‒
Femoral neck fracture	231 (62.94)	‒	‒
Intertrochanteric fracture	136 (37.06)	‒	‒
Surgical type, n (%)		‒	‒
Fixation		‒	
Simple fractures	80 (21.80)	‒	
Complex fractures	76 (20.71)	‒	
Arthroplasty		‒	
Simple fractures	112 (30.52)	‒	
Complex fractures	99 (26.98)	‒	
Preoperative complications, n (%)		‒	‒
< 2	189 (51.50)	‒	‒
≥ 2	178 (48.50)	‒	‒

BMI, Body Mass Index.

### MECP2 was decreased in patients with hip fractures and was correlated with IL-1β, IL-6, and TNF-α

**Fig. 1:**
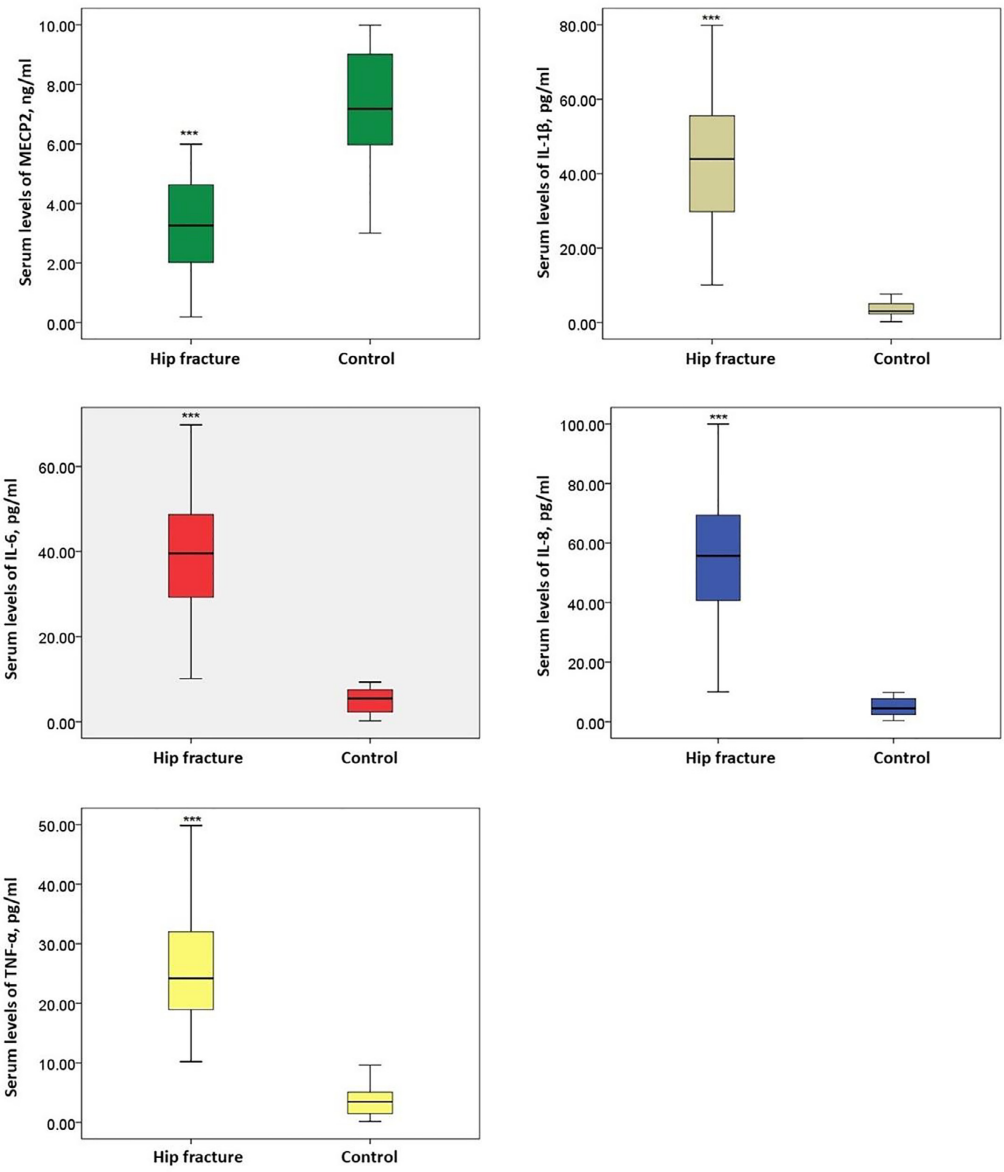
Serum MECP2 and inflammatory factor levels in patients with hip fractures and healthy controls. ***p < 0.001 vs. control. MECP2, Methyl-CpG binding Protein 2; IL, Interleukin; TNF, Tumor Necrosis Factor

**Fig. 2:**
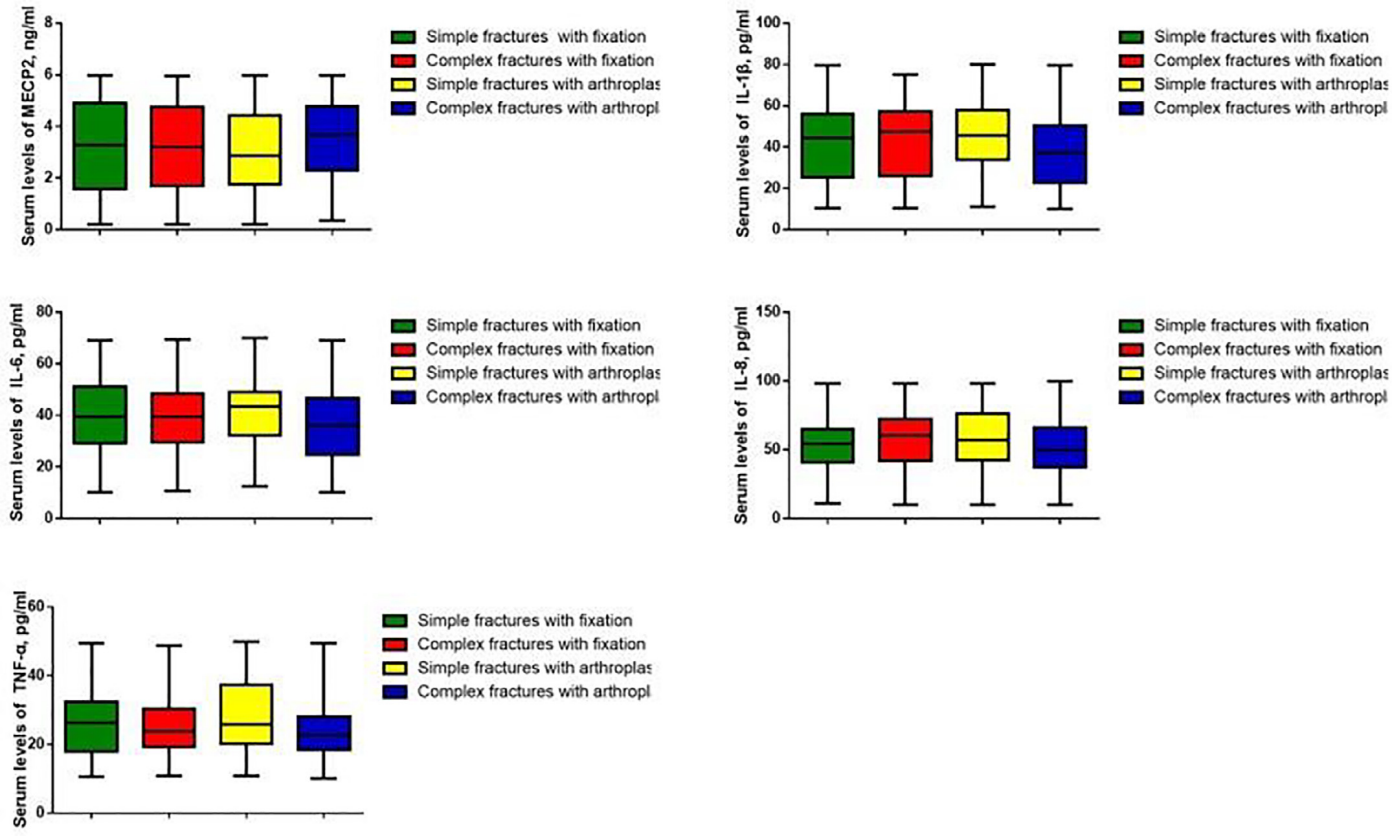
MECP2 and inflammatory factors among patients with different surgical methods. MECP2, Methyl-CpG binding Protein 2; IL, Interleukin; TNF, Tumor Necrosis Factor.

Serum MECP2 levels and inflammatory factors IL-1β, IL-6, IL-8, and TNF-α were measured. The MECP2 levels were markedly lower in patients with hip fractures than in healthy controls (p < 0.05), while the levels of IL-1β, IL-6, IL-8, and TNF-α were significantly higher in patients with hip fractures (p < 0.05) (Fig. 1). Pearson's analysis showed that MECP2 was negatively correlated with IL-1β, IL-6, and TNF-α (p < 0.05) (Table 2). However, the authors did not observe significant differences in MECP2 and inflammatory factors among patients who underwent different surgical methods (Fig. 2).

**Table 2:** Correlation among MECP2 and IL-1β, IL-6, IL-8, and TNF-α.

		MECP2	IL-1β	IL-6	IL-8	TNF-α
**MECP2**	Pearson's correlation	1	-0.654	-0.676	-0.666	-0.714
	p	‒	0.000	0.000		0.000
**IL-1β**	Pearson's correlation	-0.654	1	0.599	0.598	0.662
	p	0.000	‒	0.000	0.000	0.000
**IL-6**	Pearson's correlation	-0.676	0.599	1	0.610	0.672
	p	0.000	0.000	‒	0.000	0.000
**IL-8**	Pearson's correlation	-0.666	0.598	0.610	1	0.661
	p	0.000	0.000	0.000	‒	0.000
**TNF-α**	Pearson's correlation	-0.714	0.662	0.672	0.661	1
	p	0.000	0.000	0.000	0.000	‒

MECP2, Methyl-CpG binding Protein 2; IL, Interleukin; TNF, Tumor Necrosis Factor.

### Lower MECP2 predicted a lower Harris score, poor quality of life, and more complications

**Fig. 3:**
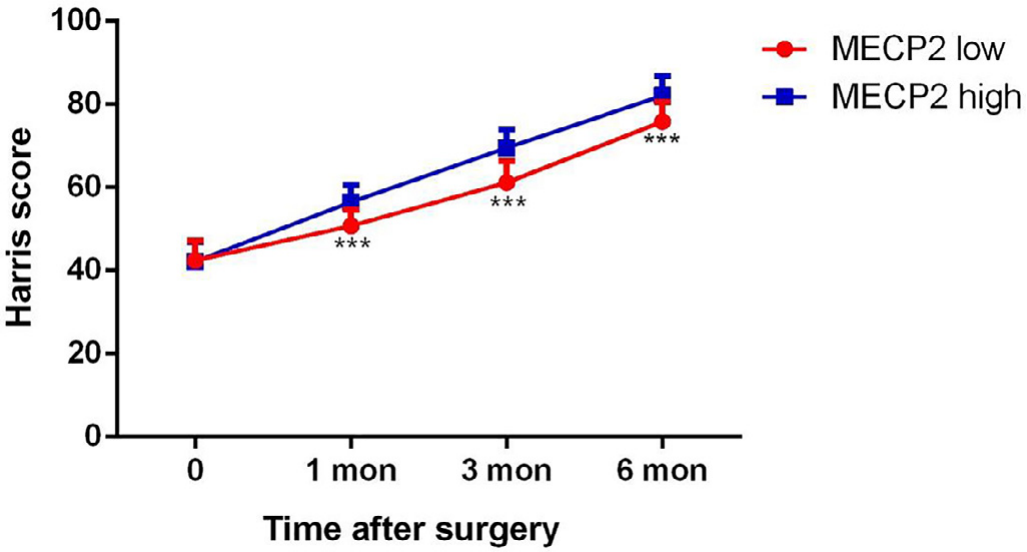
The dynamic change in the Harris score. MECP2, Methyl-CpG binding Protein 2; mon, month.

To further investigate the relationship between MECP2 and the prognosis of patients with hip fractures, the patients were divided into two groups (MECP2 high/low groups) based on the mean MECP2 value (3.26 ng/mL). The dynamic Harris score was evaluated. The hip function improved after surgery in all patients. However, patients with higher MECP2 levels showed higher mean Harris scores than those with lower MECP2 levels (p < 0.05) (Fig. 3). The BI index also showed that patients with higher MECP2 levels had higher BI values (p < 0.05) (Table 3). The total complication rate was also higher in patients with lower MECP2 levels (p < 0.05). These results indicate that MECP2 is correlated with the postoperative prognosis of patients with hip fractures.

**Table 3:** BI index 3-months after surgery and postoperative complications.

Variables	MECP2 low group, n = 184	MECP2 high group, n = 183	p
BI index	70.13±6.47	71.80±5.37	0.008
Complications, n (%)			
Poor incision healing	23 (12.50)	11 (6.01)	0.113
Respiratory complications	18 (9.78)	12 (6.56)	0.406
Cardiogenic complications	14 (7.61)	9 (4.91)	0.431
Deep vein thrombosis of the lower extremity	8 (4.35)	5 (2.73)	0.535
Urinary system complications	1 (0.54)	2 (1.09)	0.665
Total	64 (34.78)	39 (21.31)	0.034

BI, Barthel Index; MECP2, methyl-CpG binding protein 2

### Lower MECP2 predicted a higher mortality rate and shorter survival time

**Fig. 4:**
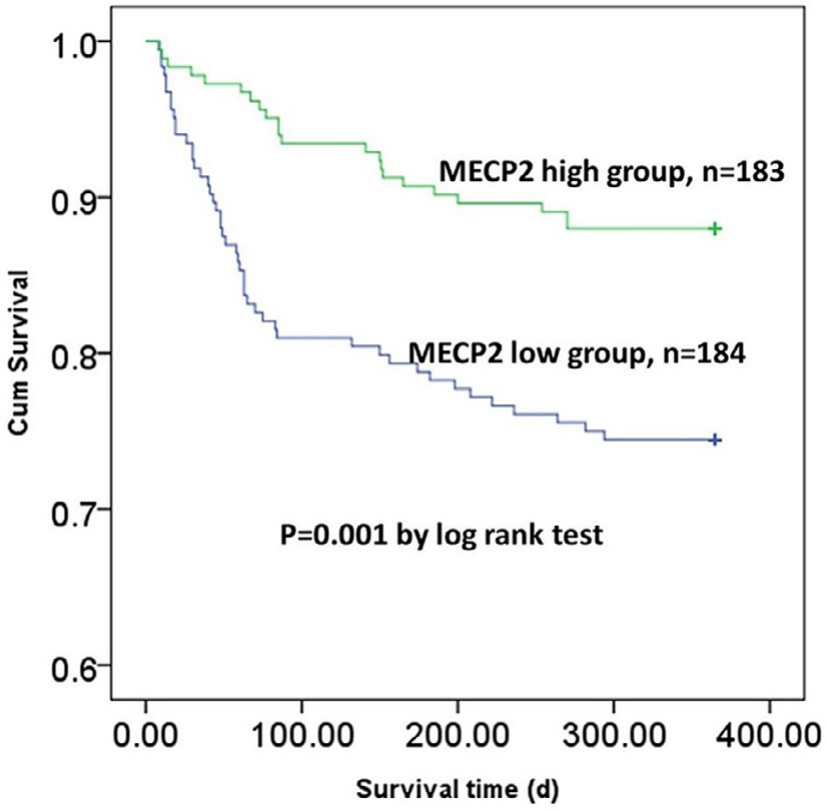
The Kaplan-Meier curve for 1-year survival. MECP2, Methyl-CpG binding Protein 2.

Finally, the authors analyzed the 1-year mortality and survival conditions of the patients. Patients with higher MECP2 had a lower mortality rate (n = 22, 12.02%) than those with lower MECP2 (n = 47, 55.95%, p < 0.05). In addition, the K-M curve also showed that patients with higher MECP2 had a remarkably longer overall survival time than those with lower MECP2 (p < 0.05) (Fig. 4). However, the logical regression showed that age was the only independent risk factor for 1-year mortality in elderly patients with hip fractures (Table 4).

**Table 4:** Independent risk factors for 1-year mortality of elderly patients with hip fractures.

	Wald	Odds ratio	95% CI	p-value
**Age**	56.400	0.202	1.224 (1.161‒1.291)	< 0.001
**BMI**	0.160	0.032	1.032 (0.881‒1.209)	0.688
**Fracture type**	0.442	-0.227	0.796 (0.407‒1.556)	0.506
**Surgical type**	0.158	-0.108	0.897 (0.526‒1.530)	0.690
**Preoperative complications**	0.483	0.348	1.417 (0.530‒3.791)	0.487
**MECP2**	11.398	0.100	0.712 (0.585‒0.867)	0.001
**IL-1β**	0.177	0.004	1.004 (0.983‒1.026)	0.673
**IL-6**	0.088	-0.004	0.996 (0.970‒1.022)	0.766
**IL-8**	0.911	0.008	1.008 (0.991‒1.026)	0.340
**TNF-α**	2.029	0.027	1.027 (0.989‒1.066)	0.154

BMI, Body Mass Index; CI, Confidence Interval; MECP2, Methyl-CpG binding Protein 2; IL, Interleukin; TNF, Tumor Necrosis Factor.

## Discussion

Hip fracture is one of the most common causes of fractures in elderly people. Despite surgical development, the mortality rate for elderly patients with hip fractures is still high; thus, new diagnosis and prognosis biomarkers may benefit patient treatment.^12^^,^^13^ In the present study, the authors demonstrated that MECP2 was downregulated in patients with hip fractures and correlated with the postoperative prognosis of elderly patients with hip fractures.

MECP2 has been shown to be associated with bone formation and related processes. It was found that a decrease in MECP2 might cause dysfunction in bone formation and reduce the bone volume in Rett's syndrome.^11^ Another study showed that MECP2 promoted fibrosis and myofibroblast trans-differentiation through an epigenetic pathway.^14^ In addition, inhibition of MECP2 might lead to suppression of osteogenic differentiation and cell apoptosis of human periodontal ligament fibroblasts.^15^ In bone recovery and wound healing, osteogenic differentiation and myofibroblast trans-differentiation are both important and may facilitate recovery duration, as well as fracture healing.^16^, ^17^, ^18^, ^19^ Thus, the authors can speculate that MECP2 may also be associated with fracture healing through the regulation of osteogenic differentiation and myofibroblast trans-differentiation. In this study, the authors observed that MECP2 was downregulated in patients with hip fractures and was correlated with patient prognosis, which supports the above speculation. However, how MECP2 influences bone formation in the recovery of hip fractures requires further investigation.

In addition to bone formation, the relationship between inflammation and MECP2 has been reported in other studies. It was considered that inhibition of MECP2 could result in the stimulation of inflammation in lung injury. On the contrary, the inflammatory response could also induce MECP2 to regulate microglial and macrophage gene expression.^20^ Li et al. demonstrated that MECP2 promotes the expression of forkhead box P3 and protects against inflammation by promoting regulatory T-cells.^21^ In addition, the lack of MECP2 also induced inflammatory factors such as IL-6 and TNF-α by activating nuclear factor-kappa B signaling.^22^ All these studies indicated that MECP2 acts as an inflammation suppressor. In the present research, the authors also found that MECP2 was negatively correlated with the inflammatory factors IL-1, IL-6, and TNF-α, which was consistent with the above studies. The stimulation of inflammatory factors is usually seen in patients with hip fractures, and higher inflammatory factors are correlated with poor prognosis.^23^, ^24^, ^25^ The authors’ results implied that MECP2 might also be involved in hip fracture promotion through regulation of inflammation, which needs to be confirmed with further studies.

## Conclusion

In conclusion, through this observational study, the authors observed that MECP2 was downregulated in patients with hip fractures, and lower MECP2 levels predicted poor prognosis in elderly patients with hip fractures. This study might provide novel insights into the role of MECP2 in hip fractures. More research is needed to further demonstrate the molecular mechanisms of MECP2 in fracture recovery and related processes.

## Authors' contributions

Gao X conducted the experiments and wrote the original manuscript draft. Xue S collected and analyzed the data. Yang F, Wu B and Yu X collected the data. An B analyzed the data and reviewed the manuscript.

## Declaration of Competing Interest

The authors declare no conflicts of interest.
